# *In vitro* anti-hepatocellular carcinogenesis of 1,2,3,4,6-Penta-O-galloyl-β-D-glucose

**DOI:** 10.29219/fnr.v67.9244

**Published:** 2023-03-27

**Authors:** Yu-han Jiang, Jing-hui Bi, Min-rui Wu, Shi-jie Ye, Lei Hu, Long-jie Li, Yang Yi, Hong-xun Wang, Li-mei Wang

**Affiliations:** 1 School of Life Science and Technology, Wuhan Polytechnic University, Wuhan, China; 2 School of Food Science and Engineering, Wuhan Polytechnic University, Wuhan, China

**Keywords:** 1,2,3,4,6-Penta-O-galloyl-β-D-glucose, apoptosis, hepatocellular carcinoma, network pharmacology, p53 signaling pathway

## Abstract

**Background:**

1,2,3,4,6-Penta-O-galloyl-β-D-glucose (β-PGG) is a polyphenol ellagic compound with a variety of pharmacological effects and has an inhibitory effect on lots of cancers.

**Objective:**

To explore the antitumor effects and mechanism of 1,2,3,4,6-Penta-O-galloyl-β-D-glucose on human hepatocellular carcinoma HepG2 cells.

**Design:**

A network pharmacology method was first used to predict the possible inhibition of hepatocellular carcinoma growth by 1,2,3,4,6-Penta-O-galloyl-β-D-glucose (β-PGG) through the p53 signaling pathway. Next, the Cell Counting Kit (CCK-8) assay was performed to evaluate changes in the survival rate of human hepatocellular carcinoma HepG2 cells treated with different concentrations of the drug; flow cytometry was used to detect changes in cell cycle, apoptosis, mitochondrial membrane potential (MMP) and intracellular Ca2+ concentration; real-time fluorescence quantification and immunoblotting showed that the expression of P53 genes and proteins associated with the p53 signaling pathway was significantly increased by β-PGG treatment.

**Reasult:**

It was found that β-PGG significantly inhibited survival of HepG2 cells, promoted apoptosis, decreased MMP and intracellular Ca2+ concentration, upregulated P53 gene and protein expression, increased CASP3 expression, and induced apoptosis in HepG2 cells.

**Conclusion:**

This study has shown that network pharmacology can accurately predict the target of β-PGG’s anti-hepatocellular carcinoma action. Moreover, it was evident that β-PGG can induce apoptosis in HepG2 cells by activating the p53 signaling pathway to achieve its anti-hepatocellular carcinoma effect *in vitro*.

## Popular scientific summary

In this paper, network pharmacology was used to predict the mechanism of action of β-PGG against hepatocellular carcinogenesis, which was verified through in vitro experiments to explore the pharmacological effects of the active ingredients in plants, and provide a basis for the research and development of new drugs.

Cancer is considered to be the greatest challenge to human life and health. Liver cancer, as the third most deadly malignancy in the world, causes about 383,000 deaths each year in China, and its late detection and poor prognosis pose a serious threat to public life and health (1–3). Studies have revealed that viral infections, metabolic diseases, and diet are important risk factors for development of liver cancer, with dietary factors leading the list (4). Consequently, prevention of cancer by food and nutrients has become a research hotspot. Over the years, a number of macronutrients, micronutrients, and non-nutrients have been reported to play an important role in cancer prevention (5), and natural ingredients derived from food have been shown to be more advantageous than synthetic compounds in the fight against tumors (6). Therefore, it is of great significance to elucidate the underlying molecular mechanism of natural ingredients against liver cancer, with the overarching goal of identifying molecular targets for the targeted therapy of liver cancer.

Polyphenols are common micronutrients in the diet and have significant therapeutic effects on degenerative diseases, such as cancer and certain cardiovascular diseases (7). 1,2,3,4,6-Penta-O-galloyl-β-D-glucose (β-PGG) is a polyphenol ellagic compound present in numerous foods, including pomegranate, rhizome, mango, and other foods with polyphenolic properties (8). It has shown strong biological and pharmacological activities in antiviral (9), anti-inflammatory (10), anti-microbial (11), and anti-diabetic (12). Previous studies have shown that β-PGG exhibits inhibitory effects on colon cancer (13), breast cancer (14), prostate cancer (15), and pancreatic cancer (16). However, there is a lack of reports on the anti-hepatocellular carcinoma effects of β-PGG and the possible mechanisms.

Network pharmacology is a drug design approach that encompasses systems biology, network analysis, connectivity, redundancy, and pleiotropy, and thus it can support the development of new drugs as well as explore biological mechanisms. Specifically, network pharmacology reveals the network of drug-gene-disease interactions, and further reflects the multi-target and multi-pathway nature of drug therapy (17).

In view of this, this study used network pharmacology to screen the possible targets of β-PGG against hepatocellular carcinoma and to systematically predict its molecular mechanism of action. We expect that this study will provide a basis for the development and application of functional foods for treating liver cancer.

## Materials and methods

### Network pharmacology experimental predictions

#### β-PGG and liver cancer target prediction and screening application

The 2D structure of β-PGG was obtained from Pubchem database, and then the sdf file of the drug structure was imported into PharmMapper and SwissTargetPrediction databases to merge and de-duplicate the drug-related targets. Next, disease related targets were obtained from the GeneCards database using the keyword ‘liver cancer’, with ‘human’ as the genus.

#### Acquisition of crossover genes and construction of protein-protein interaction (PPI) networks

The intersection of β-PGG targets with liver cancer targets was determined using JVENN software. The intersecting targets were then entered into the Protein Interaction Database (STRING) setting the species origin to human and the minimum relationship score to 0.4. Notably, the free proteins were removed to obtain the protein interactions map.

#### Acquisition of HUB genes and gene ontology and Kyoto encyclopedia of genes and genomes enrichment analyses

The protein interactions maps obtained from the STRING database were imported into Cytoscape 3.7.1 software and then the MCC calculation method in the CytoHubba plugin (18) was utilized to obtain the top eight central target proteins in the network. The central target proteins were then subjected to gene ontology (GO) and Kyoto Encyclopedia of Genes and Genomes (KEGG) analyses using the R package clusterProfiler (19). Enrichplot (20) R package was used to filter out data with *P*-value < 0.05, and finally ggplot2 (21) R package was applied to plot the relevant legends.

### In vitro *experiments*

#### Materials and reagents

Hepatocellular carcinoma cell line HepG2 was purchased from Biyuntian Biotechnology Co. Ltd.; DNA content assay kit, mitochondrial membrane potential (MMP) assay kit (JC-10), and calcium ion assay kit were supplied by Beijing Solabao Technology Co. Ltd.; T25 cell culture flasks, 96-well plates, lyophilization tubes, and six-well plates were obtained from Corning, USA; and western blot-related antibodies were purchased from Wuhan Seville Biotechnology Co.

#### Instruments and equipment

SIM CO_2_ incubator, SIM, USA; Inverted microscope, Olympus, Japan; Infinite200PRO enzyme marker, TECAN, Switzerland; Flow cytometer, Beckman coulter, USA; Thermal cycling PCR, real-time fluorescence quantitative PCR, gel imager and electrophoresis instrument, Bio-Rad, USA.

#### Cell culture

Human hepatocellular carcinoma HepG2 cells were cultured in Dulbecco's modified eagle medium (DMEM) medium supplemented with 10% fetal bovine serum to provide nutrients and 1% double antibodies to prevent contamination of the medium. Cells were grown to about 80% confluence in a 1:3 ratio of passaged culture.

#### Cell proliferation assay

Cell proliferation was detected by the Cell Counting Kit (CCK-8). Briefly, HepG2 cells were inoculated at a density of 4 × 10^4^ cells/well using complete medium in 96-well plates and incubated overnight. On the next day, the medium was aspirated and equal amounts of β-PGG (0, 12.5, 25, 50, 100, and 200 μg/mL) and 5-fluorouracil (5-FU) (positive control) were added, followed by incubation for 24 h or 48 h. Next, the medium was removed, 10 μL of CCK-8 was added to each well, and incubated at 37°C for 4 h. The optical density (OD) of each well was then measured at 450 nm by enzyme marker.

#### Cell cycle assay

Propidium iodide (PI) staining was used to detect cell cycle distribution. Briefly, HepG2 cells were digested and inoculated overnight in six-well plates until they reached 80% of the culture flask, and then they were cultured with a gradient concentration of β-PGG for 48 h. Next, trypsin digestion was performed, cell concentration was adjusted to 1 × 10^6^ cells/mL with phosphate buffered saline (PBS), and cells were fixed in 70% ethanol at -20°C overnight. On the next day, cells were collected and washed in cold PBS, and resuspended in 0.5 mL PBS. Cells were then treated with Rnase A solution for 30 min to fully degrade the RNA, followed by PI staining to determine the cell cycle phase using flow cytometry.

#### Apoptosis detection

Apoptosis was detected by the Annexin V-FITC/PI double staining method. In brief, the cell concentration was adjusted to 2 × 10^6^ cells/mL, 2 mL of the cells were inoculated in a 6-well plate, and then they were washed with PBS after 24 h and incubated with equal amounts of different concentrations of β-PGG for 48 h. After digestion with ethylene diamine tetraacetic acid (EDTA)-free trypsin, cells were collected and resuspended by adding 400 μL AnnexinV conjugate followed by 5 μL of Fluorescein isothiocyanate isomer (FITC). PI staining was then performed at 4 °C, with protection from light, and finally cell apoptosis was detected by flow cytometry.

#### Determination of MMP

The intracellular MMP was detected by JC-10 staining method. The cell concentration was first adjusted to 2 × 10^6^ cells/mL, 2 mL of the cells were then inoculated in six-well plates, followed by treating with different concentrations of β-PGG for 48 h. Next, the complete medium was adjusted to 1 × 10^6^ cells/mL and 1 mL of JC-10 staining working solution was added to stain the cells at 37°C. Finally, the stained cells were resuspended using 1× of JC-10 staining buffer and then detected by flow cytometry.

#### Intracellular calcium ion concentration assay

Cell suspensions from the passaged cultures were adjusted to a cell concentration of 2 × 10^6^ cells/mL and 2 mL of the cells was incubated in six-well plates for 24 h. Cells were then treated with different concentrations of β-PGG. After incubation for 48 h, cells were collected for subsequent probe loading operations. Finally, cells were washed and resuspended using HBSS pre-warmed to 37°C, and then detected by flow cytometry.

#### Gene expression detection by real time fluorescence quantification polymerase chain reaction

This study performed real-time fluorescence quantification of relevant genes on the pathway predicted by network pharmacology. Briefly, HepG2 cells were inoculated in culture flasks and treated for 48 h with β-PGG dissolved in complete medium to a concentration of 100 μg/mL. RNAiso Plus kit was then used to extract total RNA from HepG2 cells in accordance with the manufacturer’s instructions. Next, the extracted RNA was reverse transcribed on ice into cDNA using the Prime-script RT Master Mix kit according to the manufacturer’s protocol. Real time fluorescence quantification polymerase chain reaction (qRT-PCR) analysis was then performed using the SYBR Green Master Mix kit with the *β-actin* gene as an internal reference. (Table 1 shows sequences of the used PCR amplification primers).

**Table 1:** PCR primer sequence

Gene	Forward primer (5′→3′)	Reverse primer (5′→3′)
PUMA	GAGGAGGAACAGTGGGCC	GGAGTCCCATGATGAGATTGT
Bax	AAGAAGCTGAGCGAGTGTCT	GTTCTGATCAGTTCCGGCAC
Bcl-2	GCCTTCTTTGAGTTCGGTGG	GAAATCAAACAGAGGCCGCA
Caspase-3	ACTGGACTGTGGCATTGAGA	GCACAAAGCGACTGGATGAA
Caspase-9	GCCCCATATGATCGAGGACA	CAGAAACGAAGCCAGCATGT
P21	GACACCACTGGAGGGTGACT	CAGGTCCACATGGTCTTCCT
P53	GTTCCGAGAGCTGAATGAGG	TCTGAGTCAGGCCCTTCTGT
IGF-BP3	CCTGCCGTAGAGAAATGGAA	AGGCTGCCCATACTTATCCA
PERP	TGCCATCATTCTCATTGCAT	AACCCCAGTTGAACTCATGG
Cytochrome c	ATGAAGTGTTCCCAGTGCCA	CTCTCCCCAGATGATGCCTT
β-Actin	CATCCGCAAAGACCTGTACG	CCTGCTTGCTGATCCACATC

PCR, polymerase chain reaction.

#### Protein immunoblotting to determine protein expression

HepG2 cells were treated with 100 μg/mL β-PGG for 48 h. At the end of the treatment, cells were washed twice with TBS buffer and fully lysed with Total Protein Extraction Reagent. The cell debris was collected and the cell supernatant was collected by centrifugation at 12,000 r/min for 15 min to obtain the total protein solution. The protein concentration was determined using the BCA kit and then samples were resolved using electrophoresis. Samples were spotted on a pre-prepared gel plate, closed by transferring the membrane, incubated with antibody, and developed in a developer using the chemiluminescence Enhanced chemiluminescence (ECL) kit.

#### Statistical analyses

All statistical analyses were performed using GraphPad Prism 8.0.2 software and all data are expressed as the mean ± SD of three independent experiments. For all experiments, *P*-value < 0.05 was considered statistically significant.

## Results

### Acquisition and network construction of β-PGG targets for the treatment of hepatocellular carcinoma

**Fig. 1:**
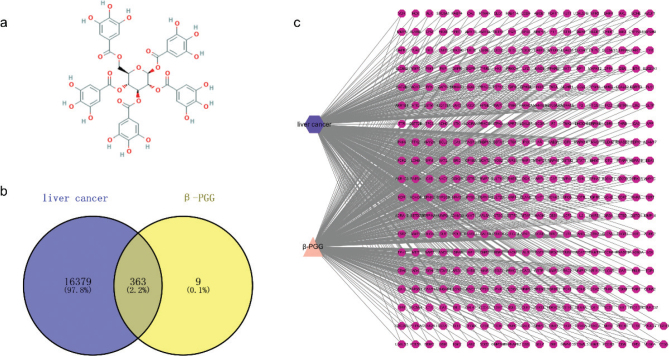
Pharmacological networks of β-PGG and liver cancer. (a) 2D structure of β-PGG. (b) Venn diagram showing the drug-disease interactions. (c) The ‘β-PGG-target-hepatocellular carcinoma’ network. β-PGG, 1,2,3,4,6-Penta-O-galloyl-β-D-glucose.

The 3D structure of β-PGG was retrieved from the PubChem database (Fig. 1a). A total of 372 genes associated with β-PGG were obtained from PharmMapper and SwissTargetPrediction databases, whereas 16,731 liver cancer-related genes were obtained from the GeneCards database. A total of 363 crossover genes were obtained after intersection of drug genes and disease genes (Fig. 1b), suggesting that β-PGG may regulate the progression of hepatocellular carcinoma through these crossover genes. To further evaluate the interrelationship between β-PGG and hepatocellular carcinoma, a ‘β-PGG-target-hepatocellular carcinoma’ network was constructed in Cytoscape 3.7.1 software (Fig. 1c).

### Construction of PPI networks and acquisition of HUB genes

**Fig. 2:**
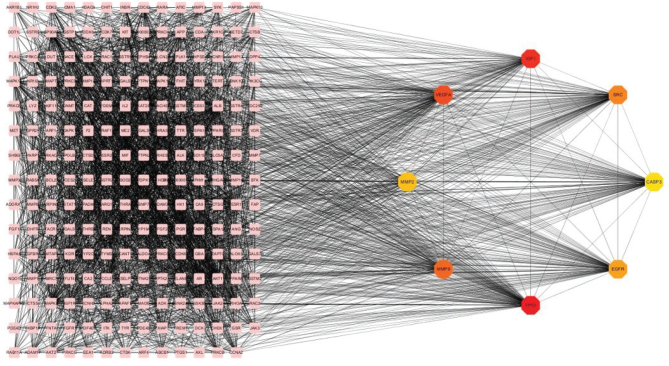
PPI network diagram and identified hub genes. PPI map showing key genes, changes are presented based on size and color of degree value. PPI, protein–protein interaction.

Cytoscape software was used to visualize the protein interactions, whereas the MCC calculation method in the CytoHubba plugin was applied to calculate the top eight target genes of the protein interactions network, namely *TP53*, *IGF1*, *EGFR*, *VEGFA*, *CASP3*, *MMP2*, *MMP9*, and *SRC* (Fig. 2). Results suggested that the above genes and related proteins play a crucial role in the liver cancer treatment.

### GO and KEGG enrichment analyses

**Fig. 3:**
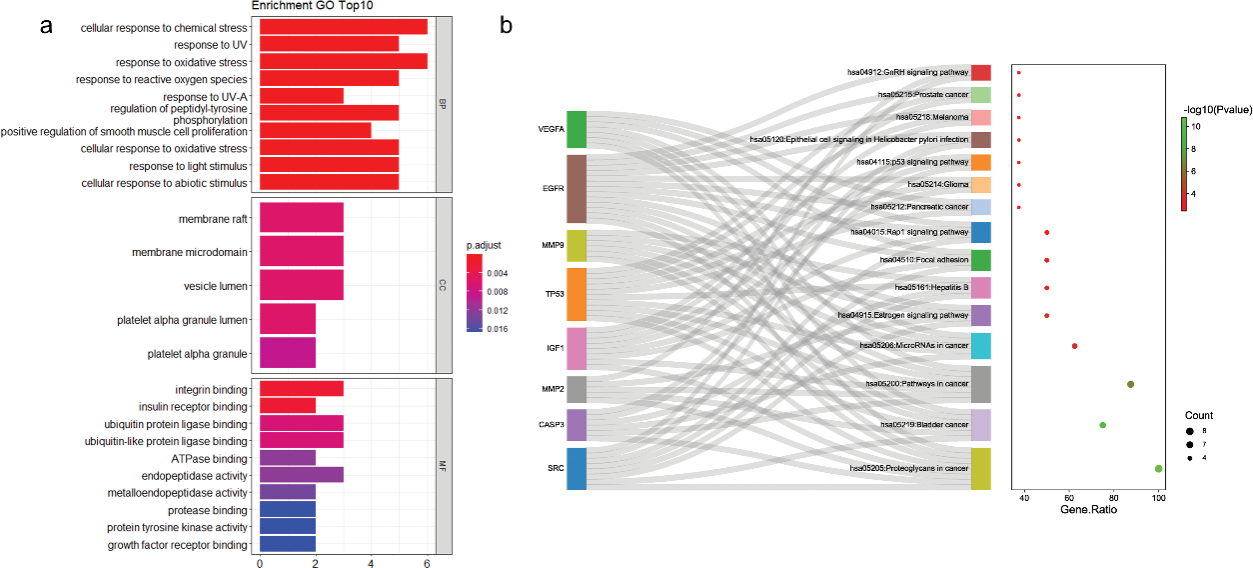
GO and KEGG pathway analysis of hub genes. (a) GO analysis. (b) KEGG pathway analysis. GO, gene ontology; KEGG, Kyoto encyclopedia of genes and genomes.

To elucidate the mechanism of β-PGG action on hepatocellular carcinoma, the eight HUB genes were subjected to enrichment analysis using R studio. Figure 3 shows the GO enrichment results which indicate that the hub genes were mainly associated with biological processes, such as negative regulation of apoptotic process, positive regulation of DNA binding, and positive regulation of mitochondrial Cytochrome c release. On the other hand, the KEGG results revealed that the genes were mainly associated with signaling pathways such as RAP1 and p53. The combined GO and KEGG analyses demonstrated that β-PGG inhibits hepatocellular carcinoma by inducing apoptosis, where *P53*, as an important oncogene, regulates the cell cycle and prevents cell carcinogenesis (22); whereas *CASP3*, as an executor of apoptosis, can remove damaged cells and catalyze cleavage of many key cellular proteins to achieve apoptosis after being activated (23). In summary, the p53 signaling pathway, which is most closely associated with apoptosis, was selected for further validation.

### Effect of different concentrations of drugs on the inhibition rate of hepatocellular carcinoma HepG2 cells

**Fig. 4:**
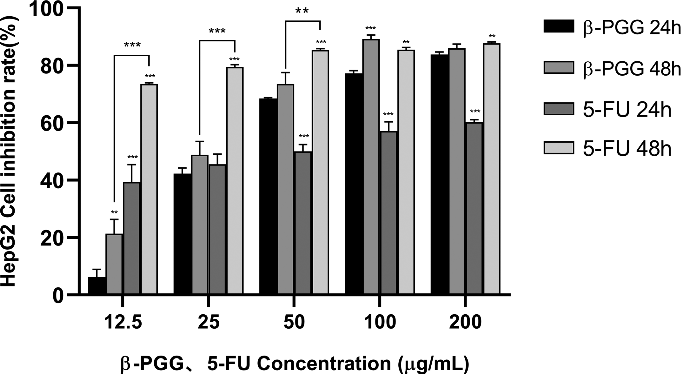
Effect of β-PGG on proliferation of HepG2 cells. HepG2 cells were treated with different concentrations of β-PGG and different concentrations of 5-FU (12.5, 25, 50, 100 and 200 μg/mL) for 24 h and 48 h, and detected by CCK-8 assay. β-PGG, 1,2,3,4,6-Penta-O-galloyl-β-D-glucose; 5-FU, 5-fluorouracil; CCK-8, Cell Counting Kit. **P < 0.01, ***P < 0.001vs. β-PGG 24 h.

Figure 4 shows that the proliferation inhibition rate of β-PGG on HepG2 cells was concentration-dependent, and the inhibition rate was positively correlated with the drug concentration. The obtained results demonstrated that the effect of β-PGG was better than that of 5-FU under the experimental conditions of drug treatment for 24 h and concentrations of 50–200 μg/mL. However, there was no significant difference between the effect of β-PGG and 5-FU under the experimental conditions of drug treatment for 48 h and concentrations of 100–200 μg/mL. The drug effects were further evaluated by calculating the median inhibition concentration (IC_50_) values of each group. Results showed that the IC_50_ of β-PGG treated HepG2 cells for 24 h and 48 h were 40.85 and 28.50, respectively, whereas the IC_50_ of 5-FU treated HepG2 cells for 24 h and 48 h were 42.95 and 21.06, respectively. Based on the IC_50_ results, it was evident that β-PGG and 5-FU were equally effective. In addition, it was found that the longer the treatment time, the stronger the killing effect on HepG2. Collectively, these results suggest that β-PGG inhibited proliferation of human hepatocellular carcinoma HepG2 cells and showed a good dose-effect relationship.

### Effect of β-PGG on the HepG2 cell cycle in hepatocellular carcinoma

**Fig. 5:**
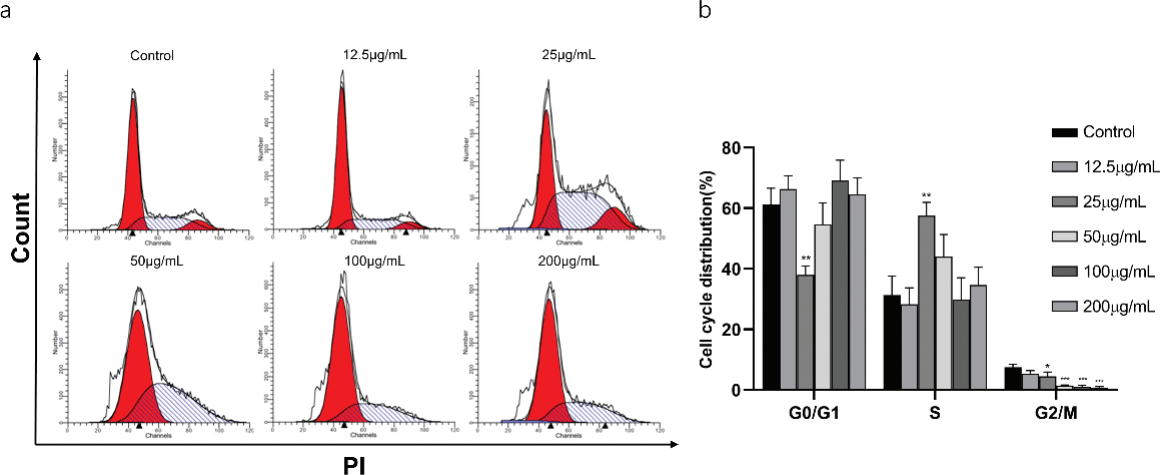
Effects of 1,2,3,4,6-Penta-O-galloyl-β-D-glucose on HepG2 cell cycle. (a) Intracellular fluorescence intensity of HepG2 cells cultured with 1,2,3,4,6-Penta-O-galloyl-β-D-glucose (0, 12.5, 25, 50, 100 and 200 µg/mL) for 48 h. (b) Average fluorescence intensity of HepG2 cells. *P < 0.05, **P < 0.01, ***P < 0.001 vs. 0 µg/mL.

Figure 5 shows that the number of hepatocellular carcinoma cells in G2/M phase decreased in a dose-dependent manner after 48 h of intervention with different concentrations of β-PGG. The proportion of S phase increased when the concentration of the drug was less than 50 μg/mL, whereas the proportion of G0/G1 phase increased when the concentration was greater than 50 μg/mL. These results suggested that the low concentration of β-PGG blocked growth of HepG2 cells in S-phase, whereas the increased concentration of the drug blocked growth of the cells in G0/G1-phase, thereby achieving the purpose of inhibiting cell growth.

### Effect of β-PGG on apoptosis of hepatocellular carcinoma HepG2 cells

**Fig. 6:**
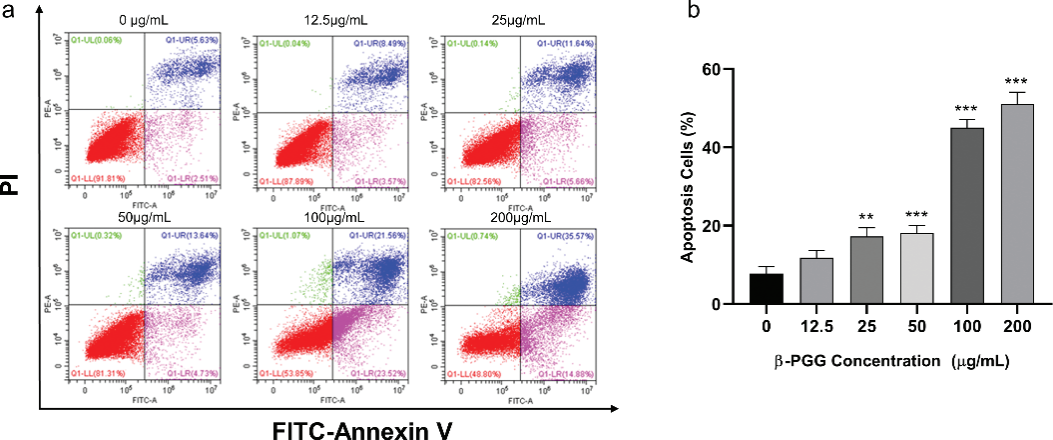
Effects of 1,2,3,4,6-Penta-O-galloyl-β-D-glucose on apoptosis of HepG2 cells. (a) FITC-Annexin V/PI double-staining flow cytometry showing apoptosis rate of HepG2 cells after treatment with 1,2,3,4,6-Penta-O-galloyl-β-D-glucose and 5-FU (0, 12.5, 25, 50, 100 and 200 µg/mL) for 48 h. (b) Average apoptosis rate of HepG2 cells. PI, propidium iodide; 5-FU, 5-fluorouracil. **P < 0.01, ***P < 0.001 vs. 0 µg/mL.

A previous study revealed that apoptosis is often associated with blockage of the tumor cell cycle (24). In the present study, the percentage of apoptotic cells increased gradually after treating for 48 h with different concentrations of β-PGG (Fig. 6). Results showed that the apoptosis rates were 36.44 and 51.02% for 100 and 200 μg/mL, respectively, which were significantly higher compared to the control group. These results suggest that β-PGG caused apoptosis in HepG2 cells.

### Effect of β-PGG on the membrane potential of mitochondria in hepatocellular carcinoma HepG2 cells

**Fig. 7:**
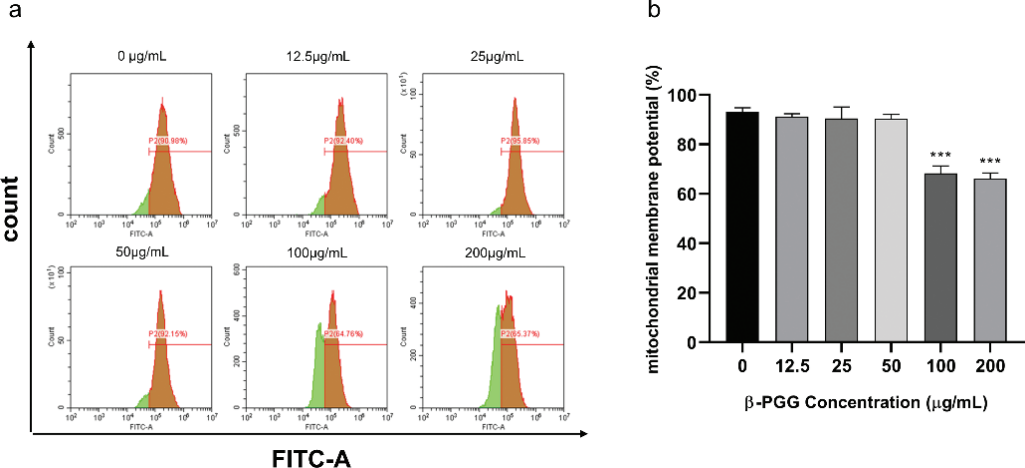
Effects of 1,2,3,4,6-Penta-O-galloyl-β-D-glucose on mitochondrial membrane potential in HepG2 cells. HepG2 cells were incubated with different concentrations of 1,2,3,4,6-Pentagram-β-D-glucose for 48 h. ∆ψm was evaluated using JC-10 in treated cells. (a) ∆ψm after treatment of cells with 0, 12.5, 50, 100, 200 μg/mL β-PGG (b) Average mitochondrial membrane potential rate of HepG2 cells. β-PGG, 1,2,3,4,6-Penta-O-galloyl-β-D-glucose. **P < 0.01, ***P < 0.001 vs. 0 µg/mL.

Mitochondria are common organelles in eukaryotes that not only power life, but also play a central role in apoptosis. Recent studies have shown that apoptosis is often accompanied by a decrease in MMP (25). Flow cytometry can show the change in red fluorescence in mitochondria, which indicates that the mitochondrial membrane is continuously disrupted. Herein, the MMP of hepatoma cells treated with different concentrations of β-PGG for 48 h changed with increasing drug concentrations, where it decreased by 25.61% at a concentration of 200 μg/mL compared to the untreated cells. The results indicated that β-PGG damaged the mitochondria and caused a decrease in the MMP of the cells (Fig. 7).

### Effect of β-PGG on intracellular calcium ion concentration in hepatocellular carcinoma HepG2 cells

**Fig. 8:**
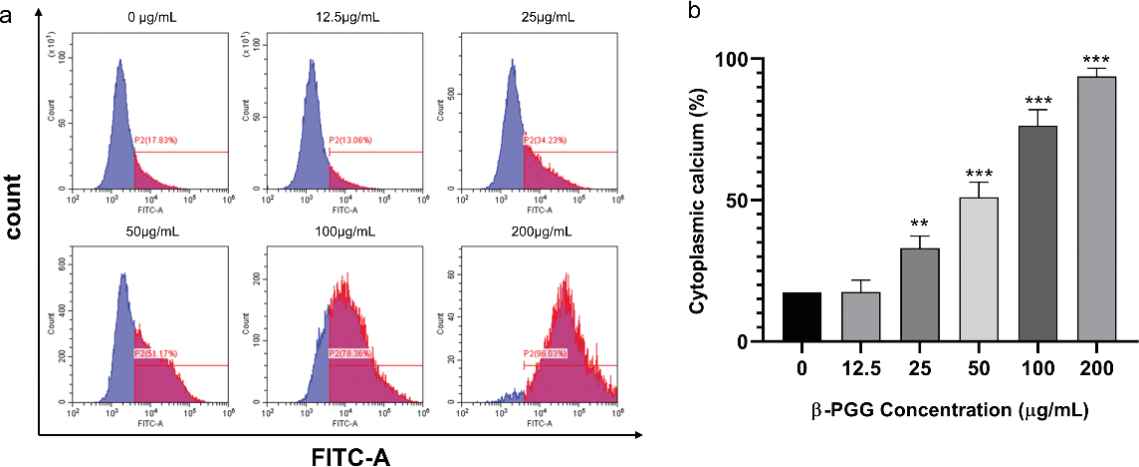
Effect of 1,2,3,4,6-Penta-O-galloyl-β-D-glucose on intracellular Ca^2+^ concentration in HepG2 cells. HepG2 cells were incubated with different concentrations of 1,2,3,4,6-Penta-O-galloyl-β-D-glucose for 48 h. Fluo 3-AM assay was performed to determine Ca^2+^ concentration. (a) Intracellular Ca^2+^ concentration after treatment of cells with 0, 12.5, 50, 100, 200 μg/mL (b) Average Cytoplasmic calcium rate of HepG2 cells. *P < 0.05, **P < 0.01, ***P < 0.001 vs. 0 µg/mL.

Excessive Ca^2+^ release can lead to disruption or even rupture of the outer mitochondrial membrane, thereby promoting release of apoptotic factors into the cytoplasm and inducing apoptosis (26). Figure 8 shows that when the cells were treated with different concentrations of β-PGG for 48 h, the intracellular calcium ion concentration increased continuously as the drug concentration increased. When the drug concentration reached 200 μg/mL, the intracellular calcium ion concentration increased significantly compared to the control group. These results suggest that β-PGG treatment leads to an imbalance of intracellular calcium ions in HepG2 cells and induces apoptosis.

### Effect of β-PGG on gene expression in hepatocellular carcinoma HepG2 cells

**Fig. 9:**
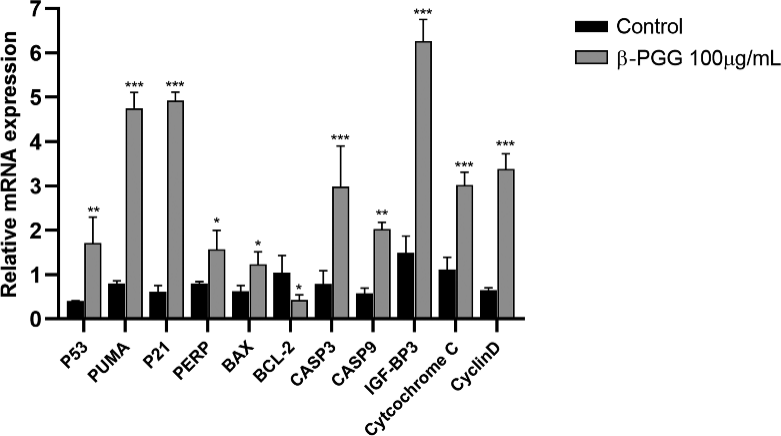
Expression levels of apoptotic-related genes in HepG2 cells. qRT-PCR was used to determine mRNA levels of apoptosis-related genes in HepG2 cells. Expression levels of *P21, PERP, IGF-BP3, PUMA, BAX, BCL-2, CASP9, Cyclin D. Cytochrome C, CASP3, P53*. Cells were treated with 100 µg/mL 1,2,3,4,6-Penta-O-galloyl-β-D-glucose for 24 h. qRT-PCR, real time fluorescence quantification polymerase chain reaction. *P < 0.05, **P < 0.01, ***P < 0.001 vs. control.

Figure 9 shows the expression of p53 signaling pathway-related genes in HepG2 cells after treatment with 100 μg/mL of β-PGG for 48 h. The expression of *P53*, *PUMA*, *P21*, *IGF-BP3*, *CASP3*, *CASP9*, *Cytochrome C,* and *CyclinD* increased significantly in the experimental group cells compared to the control group (*P* < 0.05). However, there was a decrease in the *BCL-2/BAX* ratio. These results demonstrated that the drug could induce apoptosis in hepatocellular carcinoma HepG2 cells at the genetic level by activating the p53 signaling pathway, which was consistent with the results predicted by network pharmacology.

### Effect of β-PGG on protein expression in hepatocellular carcinoma HepG2 cells

**Fig. 10:**
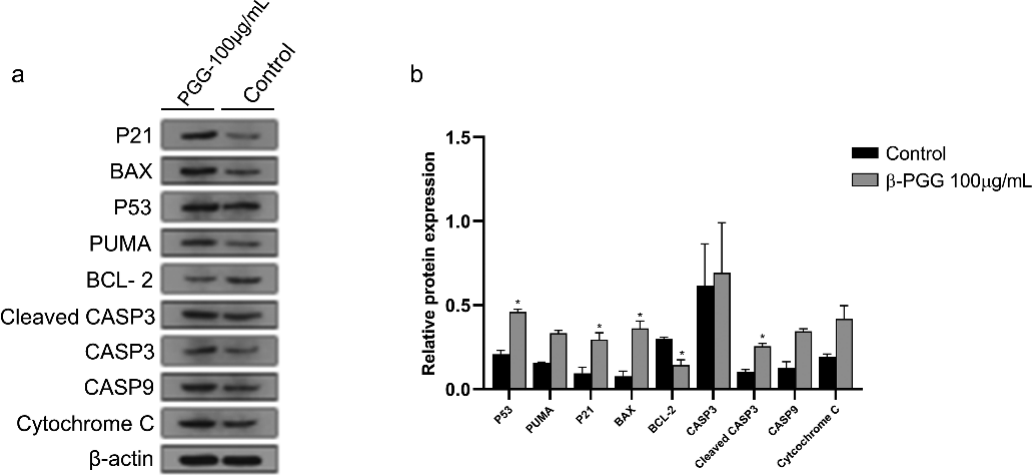
Expression levels of apoptotic-related proteins in HepG2 cells. (a) Expression levels of BAX, BCL-2, CASP3, Cleaved CASP3, CASP9, Cytochrome C, P21, P53, PUMA in HepG2 cells as determined by western blot. Cells were treated with 100 µg/mL 1,2,3,4,6-Penta-O-galloyl-β-D-glucose for 24 h. (b) The average of P21, BAX, P53, PUMA, BCL-2, PUMA, CASP3, Cleaved CASP3, CASP9 and Cytochrome C proteins band grayscale. Cells were treated with 100 µg/mL 1,2,3,4,6-Penta-O-galloyl-β-D-glucose for 24 h. *P < 0.05, **P < 0.01, ***P < 0.001 vs. control.

The expression of CASP9 and Cytochrome C proteins was upregulated in the experimental group cells compared to the control group (*P* < 0.05), whereas the expression of BCL-2 was downregulated compared to the control group (*P* < 0.05). The expression of P53, P21 and Cleaved CASP3 proteins was upregulated in the experimental group cells compared to the control group (*P* < 0.01) and the expression of BAX was upregulated in the experimental group cells compared to the control group (*P* < 0.001) (Fig. 10). This suggests that the expression of P53 protein was upregulated during the induction of apoptosis in HepG2 cells by β-PGG at 100 μg/mL. In addition, upregulation of P53 protein during the induction of apoptosis by β-PGG was shown to promote downstream expression of PUMA protein, which in turn altered MMP, activated pro-apoptotic BCL-2 family proteins, and released Cytochrome C to induce apoptosis. The results suggest that β-PGG induces apoptosis in hepatocellular carcinoma HepG2 cells at the protein level by activating the p53 signaling pathway.

## Discussion

Tannic acid is a natural hydrolyzed polyphenol. Gallic acid is a hydrolyzed tannin esterified from a glucose core to a gallic acid residue (27), having a central glucose molecule and a series of 2–12 branched gallic acid parts (27, 28). The pinwheel structure of tannic acid gives it multiple phenol groups for molecular interactions, precipitating proteins and chelating metal ions through the formation of protein complexes (29). The formation of copper complexes by tannic acid in cancer cells can generate ROS and specifically bind and disrupt DNA, leading to cell death (30). Tannic acid can also inhibit fatty acid synthetase and bind to lipids to inhibit the proliferation of cancer cells (31). 1,2,3,4,6-Penta-O-galloyl-β-D-glucose, an active substance derived from natural foods, can block the cell cycle, induce apoptosis, and exert anti-tumor effects. Studies have shown that β-PGG has a powerful cancer cell-killing effect and its effect is superior to that of gallic acid (8). Another study found that PGG blocked the cell cycle of human multiple myeloma cells RPMI8226 in the G1 phase and induced apoptosis *in vitro*. PGG can also downregulate the expression of *Cyclin D1*. A previous study revealed that low concentrations of PGG blocked the cycle of ER + breast cancer T-47D and BT-474 cells in S phase, whereas high concentrations of PGG blocked the cycle of cells in G1 phase (14). Moreover, PGG was found to downregulate the expression of HURP and BCL-2, and increase the expression of BAX to induce apoptosis as an anti-ER breast cancer (14). In colorectal cancer cells, PGG induced endogenous apoptosis by upregulating the expression of P53, P21, and cleaved CASP3. In addition, *in vivo* experiments found that PGG cured cancer cachexia by inhibiting IR and IGF1R in pancreatic cancer cells, thereby reducing glycolytic enzymes, hepatic gluconeogenesis, skeletal muscle protein hydrolysis, and fat lipolysis in tumor grafts. Dong et al. (32) reported that PGG exerted its anti-cancerous effects *in vivo* by activating MAPK8/9/10, ERN1, and EIF2S1 signaling pathways through autophagy-mediated senescence to exert its anti-hepatocellular carcinoma activity (32). However, although previous studies have shown that PGG can inhibit cancer activity, the effect of PGG on liver cancer and its potential mechanism has not yet been evaluated. This study aimed at clarifying the effect of PGG on the proliferation and apoptosis of liver cancer cells, elucidating the mechanism of action of network pharmacology, and exploring the relationship between its mechanism and the p53 signaling pathway.

Network pharmacology has the potential to expand the druggable space of proteins involved in complex diseases by mapping unexplored targets of natural products, thereby identifying new therapeutic approaches for diseases (33). In this study, 363 targets of PGG against liver cancer were identified using PharmMapper, SwissTargetPrediction, and GeneCards databases. GO and KEGG enrichment analyses revealed that PGG treatment of liver cancer was mainly associated with the p53 signaling pathway. It is worth noting that *P53* was identified as a tumor suppressor gene in 50% of human cancers in the late 1980s and 1990s. Specifically, genes with *P53* mutations were found in 50% of all human cancers (34, 35). One study revealed that *P53* is activated by various stresses to halt cancer progression by causing transient or permanent growth arrest, DNA repair, or advancing the cell death program (36).

To further validate the results of network pharmacology and explore whether PGG promotes apoptosis in hepatocellular carcinoma pairs through activation of the p53 signaling pathway, an *in vitro* hepatocellular carcinoma model was established using HepG2 cells. Results obtained after performing the CCK-8 assay showed that PGG could inhibit proliferation of hepatocellular carcinoma HepG2 cells in a time-dependent manner. Flow cytometry analysis showed that PGG blocked cell growth in the S phase and increased drug concentrations blocked cells in the G0/G1 phase. P21, the first identified CDK inhibitor, binds to cell cycle protein complexes, such as A/CDK2, E/CDK2, D1/CDK4, and D2/CDK4, thereby inhibiting phosphorylation of pRB proteins (37). It has been reported that P53 induces P21 to inhibit the cell cycle protein E/CDK2 in response to DNA damage, thereby inhibiting the G1/S transition (38). Western blot analysis showed that PGG can lead to accumulation and activation of P21.

Apoptosis occurs when internal or external factors activate the programmed cell death process. Notably, dysregulated apoptosis is a common feature of malignant tumors. In this study, flow cytometry results showed that PGG could cause apoptosis, and the apoptotic state gradually shifted toward early apoptosis as the PGG concentration increased. After combining this result with changes in MMP and Ca^2+^ concentration, we hypothesized that apoptosis was mainly endogenous. To further investigate the relationship between β-PGG inhibition of apoptosis and the p53 signaling pathway, the changes of related genes and proteins on the p53 signaling pathway were first analyzed by qRT-PCR. Results showed that the mRNA expressions of *P21*, *PUMA*, *IGF-BP3*, *CASP3,* and *Cytochrome C* genes in the cells were increased with highly significant differences (*P* < 0.01). In addition, the mRNA expression of *CASP9* and *PERP* was significantly increased (*P* < 0.05), whereas the mRNA expression of *BAX* and *P53* showed no significant difference. Notably, the expression of *BCL-2* gene was significantly decreased (*P* < 0.05). Second, western blot analysis was performed to detect the increased expression of P53, P21, Cleaved CASP3, CASP9, Cytochrome C and BAX proteins, and the decreased expression of BCL-2 protein (*P* < 0.05). Activated P53 regulated the expression of downstream P21 protein, thereby resulting in an increase in P21 protein levels in HepG2 cells with a significant difference (*P* < 0.01). Currently, it is increasingly becoming apparent that the p53 signaling pathway plays an important role in apoptosis (26), and its activation can lead to cellular angiogenesis, inhibition of apoptosis, and DNA repair, ultimately resulting in cancer development and progression.

In summary, this study has demonstrated that β-PGG achieves its anti-tumor effects *in vitro* mainly through two aspects. On one hand, it affects the cell cycle by upregulating the expression of P21 gene and protein; whereas on the other hand, it induces apoptosis in HepG2 cells by increasing the expression of P53, PUMA, and CASP9 proteins, thereby causing CASP3 to shear and its shedder content to increase the ratio of BAX to BCL-2 and promote Cytochrome C release. It is worth mentioning that the mechanisms of hepatocarcinogenesis and development are complex and thus further *in vivo* experiments are needed. Overall, this study provides more possibilities for the treatment of hepatocellular carcinoma with the help of network pharmacology and provides a reference for the development of related health food products. A preprint has previously been published (39).

## References

[1] Sung H, Ferlay J, Siegel RL, Laversanne M, Soerjomataram I, Jemal A, et al. Global cancer statistics 2020: GLOBOCAN estimates of incidence and mortality worldwide for 36 cancers in 185 countries. CA A Cancer J Clin 2021; 71(3): 209–49. doi: 10.3322/caac.21660 33538338

[2] Lv GS, Chen L, Wang HY. Research progress and prospect of liver cancer in China. Sheng Ming Ke Xue (in Chinese) 2015; 27: 237–48.

[3] Yang W-S, Zeng X-F, Liu Z-N, Zhao Q-H, Tan Y-T, Gao J, et al. Diet and liver cancer risk: a narrative review of epidemiological evidence. Br J Nutr 2020; 124(3): 330–40. doi: 10.1017/S0007114520001208 32234090

[4] Mayne ST, Playdon MC, Rock CL. Diet, nutrition, and cancer: past, present and future. Nat Rev Clin Oncol 2016; 13(8): 504–15. doi: 10.1038/nrclinonc.2016.24 26951041

[5] Di Furia L, Rusciano MR, Leonardini L, Rossi P, Giammarchi C, Vittori E, et al. A nutritional approach to the prevention of cancer: from assessment to personalized intervention. Transl Med UniSa 2015; 13: 33. 27042431 PMC4811347

[6] Tueros I, Uriarte M. Innovative food products for cancer patients: future directions. J Sci Food Agric 2018; 98(5): 1647–52. doi: 10.1002/jsfa.8789 29168190

[7] Sharma A, Kaur M, Katnoria JK, Nagpal AK. Polyphenols in food: cancer prevention and apoptosis induction. Curr Med Chem 2018; 25(36): 4740–57. doi: 10.2174/0929867324666171006144208 28990504

[8] Yang M, Memon KH, Lateef M, Na D, Wan S, Eric D, et al. 1, 2, 3, 4, 6-Pentakis [-O-(3, 4, 5-Trihydroxybenzoyl)]-α, β-D-Glucopyranose (PGG) analogs: design, synthesis, anti-tumor and anti-oxidant activities. Carbohydr Res 2016; 430: 72–81. doi: 10.1016/j.carbpol.2015.08.058 27196315

[9] Jin F, Ma K, Chen M, Zou M, Wu Y, Li F, et al. Pentagalloylglucose blocks the nuclear transport and nucleocapsid egress process to inhibit Hsv-1 infection. Jpn J Infect Dis 2016; 69(2): 135–42. doi: 10.7883/yoken.JJID.2015.137 26166506

[10] Kiss AK, Filipek A, Żyżyńska-Granica B, Naruszewicz M. Effects of penta-O-galloyl-β-D-glucose on human neutrophil function: significant down-regulation of L-selectin expression. Phytother Res 2013; 27(7): 986–92. doi: 10.1002/ptr.4822 22899541

[11] Zhao Y, Wang B, Zhang S, Yang S, Wang H, Ren A, et al. Isolation of antifungal compound from paeonia suffruticosa and its antifungal mechanism. Chin J Integr Med 2015; 21(3): 211–16. doi: 10.1007/s11655-014-1805-7 24577809

[12] Bruno E, Pereira C, Roman KP, Takiguchi M, Kao P-Y, Nogaj LA, et al. IAPP aggregation and cellular toxicity are inhibited by 1, 2, 3, 4, 6-penta-O-galloyl-β-D-glucose. Amyloid 2013; 20(1): 34–8. doi: 10.3109/13506129.2012.762761 23339420 PMC3957415

[13] Hu H, Lee H-J, Jiang C, Zhang J, Wang L, Zhao Y, et al. Penta-1, 2, 3, 4, 6-O-galloyl-β-d-glucose induces P53 and inhibits STAT3 in prostate cancer cells in vitro and suppresses prostate xenograft tumor growth in vivo. Mol Cancer Ther 2008; 7(9): 2681–91. doi: 10.1158/1535-7163.MCT-08-0456 18790750

[14] Xiang Q, Tang J, Luo Q, Xue J, Tao Y, Jiang H, et al. In vitro study of anti-ER positive breast cancer effect and mechanism of 1, 2, 3, 4-6-pentyl-O-galloyl-beta-D-glucose (PGG). Biomed Pharmacother 2019; 111: 813–20. doi: 10.1016/j.biopha.2018.12.062 30616080

[15] Holtz JN, Silverman RK, Tay KJ, Browning JT, Huang J, Polascik TJ, et al. New prostate cancer prognostic grade group (PGG): can multiparametric MRI (MpMRI) accurately separate patients with low-, intermediate-, and high-grade cancer? Abdom Radiol 2018; 43(3): 702–12. doi: 10.1007/s00261-017-1255-8 28721479

[16] Yang J, Wang F, Chen X, Qiu S, Cui L, Hu L. β-Pentagalloyl-glucose sabotages pancreatic cancer cells and ameliorates cachexia in tumor-bearing mice. Am J Chin Med 2019; 47(3): 675–89. doi: 10.1142/S0192415X19500356 30966770

[17] Hopkins AL. Network pharmacology: the next paradigm in drug discovery. Nat Chem Biol 2008; 4(11): 682–90. doi: 10.1038/nchembio.118 18936753

[18] Chin C-H, Chen S-H, Wu H-H, Ho C-W, Ko M-T, Lin C-Y. CytoHubba: identifying hub objects and sub-networks from complex interactome. BMC Syst Biol 2014; 8(4): 1–7. doi: 10.1186/1752-0509-8-S4-S11 25521941 PMC4290687

[19] Wu T, Hu E, Xu S, Chen M, Guo P, Dai Z, et al. ClusterProfiler 4.0: a universal enrichment tool for interpreting omics data. Innovation 2021; 2(3): 100141. doi: 10.1016/j.xinn.2021.100141 34557778 PMC8454663

[20] Yu G. Enrichplot: Visualization of functional enrichment result. R package version 1.10. 2[J]. Molecular Therapy: Nucleic Acids, 2021.

[21] Mailund TR. Data science quick reference, Mailund TR. Data science quick reference. Aarhus, Denmark: Springer; 2019.

[22] Lacroix M, Riscal R, Arena G, Linares LK, Le Cam L. Metabolic functions of the tumor suppressor P53: implications in normal physiology, metabolic disorders, and cancer. Mol Metab 2020; 33: 2–22. doi: 10.1016/j.molmet.2019.10.002 31685430 PMC7056927

[23] Liang Y, Yan C, Schor NF. Apoptosis in the absence of caspase 3. Oncogene 2001; 20(45): 6570–8. doi: 10.1038/sj.onc.1204815 11641782

[24] Li N, Fan L-L, Sun G-P, Wan X-A, Wang Z-G, Wu Q, et al. Paeonol inhibits tumor growth in gastric cancer in vitro and in vivo. World J Gastroenterol 2010; 16(35): 4483. doi: 10.3748/wjg.v16.i35.4483 20845518 PMC2941074

[25] Ly JD, Grubb DR, Lawen A. The mitochondrial membrane potential (∆ψm) in apoptosis: an update. Apoptosis 2003; 8(2): 115–28. doi: 10.1023/A:1022945107762 12766472

[26] Giorgi C, Baldassari F, Bononi A, Bonora M, De Marchi E, Marchi S, et al. Mitochondrial Ca2+ and apoptosis. Cell Calcium 2012; 52(1): 36–43. doi: 10.1016/j.ceca.2012.02.008 22480931 PMC3396846

[27] Enomoto H. Unique distribution of ellagitannins in ripe strawberry fruit revealed by mass spectrometry imaging. Curr Res Food Sci. 2021; 4: 821–8. doi:10.1016/j.crfs.2021.11.006 34841268 PMC8606305

[28] Watrelot AA, Le Guernevé C, Hallé H, Meudec E, Véran F, Williams P, et al. Multimethod approach for extensive characterization of gallnut tannin extracts. J Agric Food Chem 2020; 68(47): 13426–38. doi: 10.1021/acs.jafc.9b08221 32119539

[29] Falcão L, Araújo ME. Vegetable tannins used in the manufacture of historic leathers. Molecules 2018; 23(5): 1081. doi: 10.3390/molecules23051081 29751585 PMC6099987

[30] Baldwin A, Booth BW. Biomedical applications of tannic acid. J Biomater Appl 2022; 36(8): 1503–23. doi: 10.1177/08853282211058099 34991392

[31] Nagesh PKB, Chowdhury P, Hatami E, Jain S, Dan N, Kashyap VK, et al. Tannic acid inhibits lipid metabolism and induce ROS in prostate cancer cells. Sci Rep 2020; 10(1): 980. doi: 10.1038/s41598-020-57932-9 31969643 PMC6976712

[32] Dong Y, Yin S, Jiang C, Luo X, Guo X, Zhao C, et al. Involvement of autophagy induction in penta-1, 2, 3, 4, 6-O-galloyl-β-D-glucose-induced senescence-like growth arrest in human cancer cells. Autophagy 2014; 10(2): 296–310. doi: 10.4161/auto.27210 24389959 PMC5396085

[33] Kibble M, Saarinen N, Tang J, Wennerberg K, Mäkelä S, Aittokallio T. Network pharmacology applications to map the unexplored target space and therapeutic potential of natural products. Nat Prod Rep 2015; 32(8): 1249–66. doi: 10.1039/C5NP00005J 26030402

[34] Olivier M, Hollstein M, Hainaut P. TP53 mutations in human cancers: origins, consequences, and clinical use. Cold Spring Harb Perspect Biol 2010; 2(1): a001008. doi: 10.1101/cshperspect.a001008 20182602 PMC2827900

[35] Robles AI, Harris CC. Clinical outcomes and correlates of TP53 mutations and cancer. Cold Spring Harb Perspect Biol 2010; 2(3): a001016. doi: 10.1101/cshperspect.a001016 20300207 PMC2829964

[36] Stegh AH. Targeting the P53 signaling pathway in cancer therapy–the promises, challenges and perils. Expert Opin Ther Targets 2012; 16(1): 67–83. doi: 10.1517/14728222.2011.643299 22239435 PMC3291789

[37] Choi W-I, Kim M-Y, Jeon B-N, Koh D-I, Yun C-O, Li Y, et al. Role of promyelocytic leukemia zinc finger (PLZF) in cell proliferation and cyclin-dependent kinase inhibitor 1A (P21WAF/CDKN1A) gene repression. J Biol Chem 2014; 289(27): 18625–40. doi: 10.1074/jbc.M113.538751 24821727 PMC4081908

[38] Karimian A, Ahmadi Y, Yousefi B. Multiple functions of P21 in cell cycle, apoptosis and transcriptional regulation after DNA damage. DNA Repair 2016; 42: 63–71. doi: 10.1016/j.dnarep.2016.04.008 27156098

[39] Jiang Y, Bi J-H, Wu M-R, Ye S-J, Yi Y, Wang H-X, et al. In vitro anti-hepatocellular carcinogenesis of 1,2,3,4,6-penta-O-galloyl-β-D-glucose. 2022, PREPRINT (Version 1) available at Research Square. doi: 10.21203/rs.3.rs-1645156/v1 PMC10084503 37050924

